# Tumor Microenvironment in Metastatic Colorectal Cancer: The Arbitrator in Patients’ Outcome

**DOI:** 10.3390/cancers13051130

**Published:** 2021-03-06

**Authors:** Cristina Galindo-Pumariño, Manuel Collado, Mercedes Herrera, Cristina Peña

**Affiliations:** 1 Medical Oncology Department, Ramón y Cajal University Hospital, IRYCIS, CIBERONC, Alcalá University, 28034 Madrid, Spain; crisgpuma@gmail.com (C.G.-P.); manualmansa98@gmail.com (M.C.); 2 Department of Oncology-Pathology, Karolinska Institutet, 17164 Stockholm, Sweden

**Keywords:** colorectal cancer, liver metastases, tumor microenvironment, biomarkers, liquid biopsy

## Abstract

**Simple Summary:**

Colorectal cancer accounts for approximately 10% of all annually diagnosed cancers worldwide being liver metastasis, the most common cause of death in patients with colorectal cancer. The interplay between tumor and stromal cells in the primary tumor microenvironment and at distant metastases are rising in importance as potential mechanisms of the tumor progression. In this review we discuss the new biomarkers derived from tumor microenvironment and liquid biopsy as emerging prognostic and treatments response markers for metastatic colorectal cancer. We also review the developing new clinical strategies based on tumor microenvironmental cells to tackle metastatic disease in metastatic colorectal cancer patients.

**Abstract:**

Colorectal cancer (CRC) is one of the most common cancers in western countries. Its mortality rate varies greatly, depending on the stage of the disease. The main cause of CRC mortality is metastasis, which most commonly affects the liver. The role of tumor microenvironment in tumor initiation, progression and metastasis development has been widely studied. In this review we summarize the role of the tumor microenvironment in the liver pre-metastatic niche formation, paying attention to the distant cellular crosstalk mediated by exosomes. Moreover, and based on the prognostic and predictive capacity of alterations in the stromal compartment of tumors, we describe the role of tumor microenvironment cells and related liquid biopsy biomarkers in the delivery of precise medication for metastatic CRC. Finally, we evaluate the different clinical strategies to prevent and treat liver metastatic disease, based on the targeting of the tumor microenvironment. Specifically, targeting angiogenesis pathways and regulating immune response are two important research pipelines that are being widely developed and promise great benefits.

## 1. Introduction

In colorectal cancer (CRC), mortality rates vary widely, depending on the stage of the disease. The main cause of CRC mortality is metastasis, with a five-year survival rate of approximately 10% for stage IV disease [1,2]. The process of metastasis requires invasion of a secondary tissue and cell growth. This process is relatively inefficient, as only 0.01% of circulating tumor cells cause successful metastasis [3]. 

In patients with metastatic CRC (mCRC), the liver is the most common site for metastasis. Around 20–30% of CRC patients present with hepatic metastasis at diagnosis, and 50–60% will develop it during the course of the disease [4]. This high incidence is due in part to anatomical distribution. The portal vein and hepatic artery supply blood to the liver; and cancer cells disseminating from the colon easily access the liver through the portal vein [5]. Primary CRC tumor location affects metastasis sites, with liver metastasis more common in left-sided CRC. However, it has been observed that right-sided CRC patients with liver metastasis have a higher mortality risk, due in part to the presence of a higher Tumor, Node, Metastases (TNM) stage at diagnosis [6].

### 1.1. Microenvironment in mCRC

It is known that tumor microenvironment (TME) plays a crucial role in tumor initiation and progression. The “seed and soil” hypothesis proposed by Paget et al. suggests that tumor cells (*seeds*) travel to distant sites (*soil*) where the TME was favorable to colonization [7,8].

**Figure 1:**
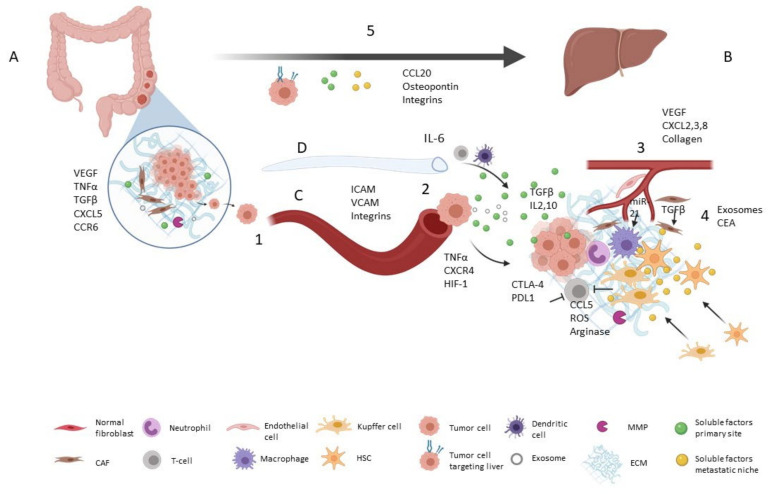
Pre-metastatic niche promotion by tumor microenvironment. Primary tumor (**A**) secretes soluble factors to prepare pre-metastatic niche (**B**). (1) Tumor cell leaves primary site, moving across extracellular matrix (ECM) and reaching blood stream (**C**) or lymphatic vessel (**D**) [2,8,13]. (2) Extravasation is mediated by vascular endothelial cell receptors and tumor cell adhesion molecules (Intercellular Cell-Adhesion Molecule (ICAM), Vascular Cell-Adhesion Molecule (VCAM)) [6]. Moreover, levels of TNFα, C-X-C chemokine Receptor type 4 (CXCR4) and Hypoxia-inducible Factor 1 (HIF-1) act in extravasation [3,6,8]. (3) Presence of VEGF, CXCL2-3-8 promotes niche vascularization [11,14]. Collagen provides scaffold for new vessels [5]. (4) In premetastatic niche, tumor-derived secreted factors recruit Kupffer cells, hepatic stellated cells and neutrophils [5]. Niche ECM is remodeled by local fibroblasts and MMPs [11]. Soluble factors TGF-β, IL2, IL10 and tumor inhibitory molecules Cytotoxic T-Lymphocyte Antigen 4 (CTLA-4) and Programmed Death-ligand 1 (PD-1), promote immunosuppression [5]. (5) Tumor cells and pre-metastatic niche itself secrete factors that drive tumor cells to liver (i.e., C-C Motif Chemokine Ligand 20 (CCL20), CXCR4, Carcinoembryonic antigen (CEA), osteopontin) [3,15,16,17]. Moreover, tumor cell surface receptors condition distal organ targeting [15,16]. Created with BioRender.com (accessed on 1 May 2020).

Primary tumor prepares distant niches by releasing tumor-derived secreted factors, including pro-angiogenic factors (e.g., Vascular Endothelial Growth Factor (VEGF)) and pro-inflammatory factors (e.g., Tumor Necrosis Factor α (TNFα), Transforming Growth Factor β (TGF-β), Interleukins (ILs)) [9,10]. Liberation of chemokines such as C-X-C motif chemokine 5 (CXCL5) and C-C Motif Chemokine Receptor 6 (CCR6) by primary tumor is associated with liver metastasis and worse prognosis [11]. Tumor-derived secreted factors promote the recruitment of Kupffer cells, hepatic stellate cells, myeloid-derived suppressor cells and neutrophils in the pre-metastatic niche [5]. Hepatic stellate cell activation plays a crucial role in liver TME remodeling by secreting growth factors (TGF-β, Epidermal Growth Factor (EGF), VEGF, Insulin-like Growth Factor (IGF)) and metalloproteinases (MMP-2, -9, -13) [11]. Changes in CRC initiating cells’ metabolism also condition liver metastasis. Increased lysine catabolism in these tumor-initiating cells Cluster of Differentiation 110+ (CD110+) activates glutamate generation and drives liver metastasis [12] (Figure 1 and Table 1).

This pre-metastatic niche preparation can be separated into different phases:

#### Extravasation and Angiogenic Process

First of all, the CRC cells have to lose epithelial properties in order to migrate, a mechanism known as epithelial-mesenchymal transition. Then, tumor cells migrate to vessels moving across the extracellular matrix that has previously been modified, mainly by cancer associated fibroblasts (CAFs) and MMPs’ enzymatic action [2,13]. These MMP levels can be regulated by interleukins and TNF-α [15]. 

### 1.2. Extravasation

Migration of tumor cells to blood vessels and extravasation are complex processes with many components involved. Vascular endothelial cell receptors from the selectin family are involved in cell-cell adhesion and transendothelial migration [22]. Tumor cell secretion of cytokines increases the expression of cell-adhesion molecules (CAM) in endothelial cells, such as ICAM and VCAM [6]. Integrins also mediate in cell adhesion via CAM molecules. Transmigration into the extravascular space is thought to be mediated by TNF-α and ICAM interaction between tumor and endothelial cells [6]. High levels of molecules like HIF-1 and CXCR4 have been studied as promoters of extravasation of cancer-circulating cells [3,8].

### 1.3. Angiogenesis

Angiogenesis and vascular permeability are also necessary steps in the process of metastatic niche preparation. The liver is a highly irrigated organ, processing 27% of cardiac output and facilitating the use of host vasculature by tumor-colonizing cells [11]. However, neovascularization is important to maintain nutrient and oxygen supply in the tumor. Under hypoxia, proangiogenic factors such as VEGF increase and endothelial progenitor cells are recruited [8]. Depending on VEGF isoform levels, there is activation of endothelial cell migration and differentiation or vascular permeability increase, conditioning the angiogenesis process [15]. 

Moreover, chemokines CXCL2, CXCL3 and CXCL8 promote tumor vascularization [11,14]. In response to inflammatory cytokines, collagen deposit types I and IV increase, providing the scaffold for endothelial cells and vessel formation [5].

#### 1.3.1. Immune Surveillance Evasion

Circulating tumor cells can activate a cytotoxic T-cell response in the liver, promoting immune surveillance evasion via inhibitory molecules such as CTLA-4 or PD-1 [5]. Among bone marrow-derived dendritic cells (BMDCs), myeloid-derived suppressor cells (MDSCs), including granulocytes, monocytes, dendritic cells as well as immature precursors, may negatively regulate cytotoxic T-cell population, playing a role in immunosuppression [11].

In addition, local cells, such as Kupffer and hepatic stellate cells, are involved in MDSC recruitment by CXCL1-2 release and T-cell inhibition mediated by CCL5, Reactive Oxygen Species (ROS) and arginase production [5]. 

The dual role of neutrophils in recruitment to the pre-metastatic niche has been reported. Neutrophils act against tumor growth by secreting cell-killing species that attack tumor cells and by recruiting host immune cells. They also play a role in matrix remodeling by secreting MMPs, so facilitating tumor cell migration and tumor growth [11].

Other factors such as TGF-β, IL2, IL10 and CTLA-4 are involved in tumor-associated macrophage polarization to the M2 phenotype, as well as naïve T-cell polarization into inducible T-reg. Both polarizations are associated with immunosuppression [5].

Tumor cells can also reach the liver via lymphatic vessels [8]. Tumor-derived VEGF-A, -C and -D stimulate lymphangiogenesis via endothelial cells’ VEGFR3 and are associated with higher incidence of liver metastasis [3,8,18]. Extravasation from lymphatic vessels can also be conditioned by tumor-derived secreted factors like IL-6, which promotes CCL5 and VEGF expression [8,20]. As one of the main roles of lymph vessels is immune-cell transport, lymphangiogenesis in pre-metastatic niche facilitates the immunosuppression process by recruiting immature DCs and T-cells [8]. Immature dendritic cells (DCs) exposed to high levels of VEGF-A have been shown to stimulate T cells to an immunosuppressive phenotype [23].

#### 1.3.2. Organotropism and Tumor Growth

As has been commented above, CRC has organotropism to liver invasion. Tumor cell-derived factors and tumor cell-surface receptors drive tumor cells to pre-metastatic niches. Moreover, pre-metastatic niches themselves secrete factors to attract circulating tumor cells. Thus, chemokine secretion by both tumor and distal organs condition metastasis. Under inflammatory conditions, chemokine CCL20, the ligand for the CCR6 receptor, which is associated with the attraction of CCR6+ tumor cells, is upregulated to liver [16].

Levels of CXCR4, associated with liver metastasis and involved in tumor expansion, are related to poor prognosis [3]. Moreover, the Carcinoembryonic antigen, widely studied in relation to CRC and liver metastasis and which is normally present in liver cells, increases retention of metastatic cells in the liver and adapts the hepatic environment to permit CRC cells to survive [17]. In a similar way, CRC osteopontin expression in colon primary tumor tissue is related to liver metastasis [15]. As for surface receptors, it has been reported that specific integrin content (α6β1, α6β4 and/or α2β1) in CRC cells confers preferential binding on hepatic vessels and hepatic resident cells [15].

Following colonization, metastatic tumor success depends on further tumor growth, which requires modification of the liver structure. Metastasis usually progresses within the liver by transforming the local stroma [24]. Malignant cell colonization of pre-metastatic niches promotes further tumor cell arrival, increasing tumor mass and causing detectable macrometastasis [8]. Sometimes, tumor cell dormancy is a response after surgery or chemotherapy, while local ECM remodeling and immunosuppression may activate tumor cells. In this scenario, the regulatory role of some micro-RNAs (miRNAs) expression in tumor cell dormancy until the pre-metastatic niche is prepared for cell growth has been reported [8].

### 1.4. Preparation of the Pre-Metastatic Niche in Secondary Organs. Exosomes’ Role 

The metastasis event needs the preparation of a favorable pre-metastatic niche, involving local stromal cells, BMDCs, immune cells, tumor-derived secreted factors and extracellular vesicles [8,9].

Exosomes are extracellular vesicles (40–150 nm) that contain mainly non-coding RNAs, but also proteins, lipids, small DNAs and messenger RNA (mRNA) [25,26]. Exosomes can be found in biological fluids like blood, and many studies focus on detecting these vesicles and identifying them by means of liquid biopsy as specific disease biomarkers [27]. In the cancer context, it was observed that cancer cells secrete more exosomes than normal cells [10]. Moreover, the cargo of tumor-derived exosomes varies over time, reflecting both the spatial and temporal heterogeneity of the disease [28]. Several studies have shown that the cargo of CRC cell line-derived exosomes varies, depending on whether the primary tumor is at early or late stage [29]. Exosomes derived from tumor cells can reach the pre-metastatic niche and participate in its transformation before the arrival of CRC cells [8]. TME acidification by exosomes plays an important role in pre-metastatic niche preparation [30]. Moreover, exosomes act in BMDC recruitment to both the primary tumor and metastatic niche [31]. Exosomes isolated from the culture medium of CRC-cell lines have been reported to contain molecules involved in pro-tumorigenic events. For instance, SW403-derived vesicles contain Fas ligand and TNF-Related Apoptosis Inducing Ligand (TRAIL) (apoptosis-related molecules); SW480 vesicles enclosed tumor susceptibility gene 101 (Tsg101) and CEA(involved in morphological changes); HT29-19 exosomes contain major histocompatibility complex (MHC) class I molecules (immune system modulation); HCA-7 cell-derived exosomes contain EGFR ligand-heparin-binding (related to invasiveness) reviewed by Uddin S and collaborator [2]. Exosomes can also release MMPs and other pro-invasive regulators [2]. Similarly, CRC cell-derived exosomes contain CEA and added to normal colonic cells, induce malignization [13]. 

Exosome membrane integrins condition liver tropism. Integrins αvβ6 and αvβ5 drive exosomes to liver, where vesicles finally fuse with Kupffer cells [10,21]. Similarly, pancreatic cancer-derived exosomes fuse with Kupffer cells [9]. Moreover, exosome membrane proteins can be transferred from primary tumor to target cells in the metastatic niche. Transfer of EGFR to liver cells and activation of Hepatocyte Growth Factor (HGF) signaling pathways has been reported [32].

A very well-known component of tumor cell-derived exosomes are miRNAs, which also play a role in pre-metastatic niche preparation. The role of exosomal miR-21, which activates polarization of macrophages to M2 phenotype, producing pro-inflammatory IL-6 and finally promoting liver metastasis, has also been studied [33].

Tumor-derived exosomes are related to immune surveillance because they both recruit immune cells and contain immunosuppressive molecules. Thus, they are involved in Treg expansion and inhibition of natural killer cells (NKs) and DCs, contributing to an immunosuppressive environment [10]. Moreover, PDL-1 exosomes induce CD8+ T cell suppression [9]. 

In addition, TGF-β contained in tumor-derived exosomes activates transformation of normal quiescent fibroblasts into cancer-associated fibroblasts, which will modify TME in pre-metastatic niche [10,19].

## 2. Prognostic and Predictive Biomarkers in the TME and the Liquid Biopsy of mCRC

Genetic alterations in tumor cells and clinicopathological biomarkers may provide prognostic and predictive information for mCRC patients. These genomic alterations include Kirsten rat sarcoma viral oncogene (*KRAS*) and neuroblastoma rat sarcoma viral oncogene (*NRAS*) mutations, type B rapidly accelerated fibrosarcoma gene (*BRAF*) mutations, *human EGF receptor 2 gene* (*HER2*) amplifications, deficient mismatch repair (dMMR) or microsatellite instability-high (MSI-H), neurotrophic receptor tyrosine kinase (*NTRK*) fusions, phosphatidylinositol 3-kinase catalytic subunit alpha (*PIK3CA)* mutations and *MET* amplification [34,35]. Currently, only rat sarcoma oncogene (*RAS)* mutation status is routinely used as a negative predictive marker to avoid treatment with anti-EGFR agents in patients with mCRC; and mismatch repair (MMR) status may guide the use of immune checkpoint inhibitors [36]. Primary tumor location and primary tumor resection might also be important prognostic parameters for metastatic CRC patients [35,36]. In addition, a considerable number of studies have evaluated the prognostic and predictive capacity of alterations in the stromal compartment of tumors (Table 2). Moreover, analysis of liquid biopsy biomarkers has emerged as a potential tool in the management of patients with mCRC (Table 3). We describe the role of tumor microenviroment cells and related liquid biopsy biomarkers in precision medicine for mCRC.

### 2.1. Cancer-Associated Fibroblasts

CAFs constitute a prognosis-associated heterogeneous cell population which have shown to exert multiple regulatory functions on tumor cells and TME components. The difficulty in the definition of these cells results largely from the lack of unique markers that are not expressed in any other cell types. Nevertheless, there are commonly recognized CAF markers proteins, still under debate, like alpha-smooth-muscle actin (aSMA), fibroblast-activated protein (FAP), fibroblast-specific protein-1 (FSP1/ S100A4) and Platelet-derived growth factor (PDGF) receptors reviewed in [101,102] which have shown prognostic relevance in different tumor types including CRC [37,38,39]. Moreover, the emergence of single cell technologies together with multi-marker fluorescence-activated cell sorting (FACS) approaches and others functional assays have reported the existence of tentative CAF subsets which shed light on the issue of the CAF heterogeneity [103,104,105].

#### 2.1.1. Prognostic Value

In stage II CRC patients, expression of endoglin in the invasion front is associated with poor metastasis-free survival. In addition, endoglin-CAF expression is found in lymph node and liver metastasis, which suggests its role in cancer metastasis [40]. Moreover, certain CAF biomarkers in liver metastases are associated with worse outcome of CRC patients. Thus, CXCL1 expression in epithelial and CAF cells or loss of phosphatase and tensin homologue (PTEN) expression in metastatic CAFs are associated with decreased overall survival in mCRC patients [41,42]. In addition, the downregulation of miR-198 in the tumor stroma of CRCs with synchronous liver metastasis is associated with Tenascin C upregulation promoting liver metastasis. Thus, Tenascin C in primary CAFs could be a novel biomarker to predict metastasis prognosis [43]. In fact, Tenascin C expression was determined as an independent marker for poor prognosis of CRC due to its promotion of EMT-like changes and proliferation [44].

In addition, some studies have described CAF gene expression signatures associated with CRC metastasis formation [106,107]. In parallel, a specific proteomic signature of extracellular matrix proteins has been observed in liver metastases, as against normal liver samples, indicating their potential use as diagnostic and prognostic tools [45]. 

#### 2.1.2. Predictive Value

Expression of claudin-2 in CAFs from primary tumors of mCRC patients is associated with shorter progression-free survival in those patients that received 5-fluorouracile (5-FU) + oxaliplatin combination treatment than in those that received 5-FU+irinotecan [46].

The most common targeted therapies in metastatic CRCs are EGFR blocking antibodies [34,108]. Thus, mutations in CAF markers such as Fibroblast growth factor receptor 1 (FGFR1) or PDGFRα, as well as consensus molecular subtypes 4 (CMS4) mesenchymal tumors are involved in resistance mechanisms to this therapy [47,48,49]. Similarly, FGFR2 amplification was a predictive marker of regorafenib sensitivity [50]. Finally, an interactome signature was observed by next generation sequencing (NGS) in relapsed and refractory mCRC patients, in which the FGFR pathway was also observed as one of the most frequently activated signaling pathways [51].

#### 2.1.3. Challenges, Limitations and Future Direction of CAF-Related Biomarkers in mCRC

Although many biomarkers related with CAFs were described in several studies in mCRC patients, the huge inter- and intra-tumor heterogeneity restricts the translation of these potential tools to clinical care of the patients. Understanding the genomic and transcriptomic heterogeneity of CAFs subtypes and identifying those with significant biological characteristics by clinical trials that include a large number of patients, will lead to an integrated approach “multi-markers—drug combination” rather than the preexisting paradigm based on “one marker—one drug” [109]. Under this scenario, clinical trials may be designed by molecular characteristics to drive therapies based on different biomarkers and to correctly select the candidate patients.

### 2.2. Endothelial Cell-Related Markers

Various studies point to the predictive value of some specific markers in peripheral blood, such as changes in VEGF pathway (increase in VEGF expression, decrease of VEGFR-2 or polymorphism in different components of the pathway), decrease of circulating endothelial cells and alterations in microvessel density [110]. Moreover, the concentration of several angiogenic factors in serum or tissue are associated with mCRC progression [111]. These factors are, therefore, currently studied as potential biomarkers in mCRC.

For many years microvessel density has been seen as a biomarker of poor prognosis in colorectal cancer. Its analysis can help in treatment decision-making, specifically regarding bevacizumab application [52]. Recently, in a clinical phase II study the association of low microvessel density with reduced progression-free survival in mCRC was confirmed [53].

As was expected, different components of the VEGF pathway have been proposed as predictive biomarkers of bevacizumab treatment in mCRC. Thus, overexpression of VEGF and specifically VEGF-A are associated with better overall and progression-free survival in bevacizumab-treated patients [54,55,56]. The role of the VEGFA145b isoform in resistance to bevacizumab was also found: it showed an interesting interaction that depended on tumor location. Thus, expression of this isoform was favorable in patients with left-side tumors, but unfavorable in those with right-side tumors [57]. Finally, the downregulation of the Neurogenic locus Notch Homolog protein 1 (NOTCH1) receptor was also associated with progression-free survival improvement in bevacizumab-treated mCRC patients [58].

Interestingly, a VEGF and VEGFR genotyping analysis in tumor and blood samples of 138 regorafenib-treated mCRC patients showed that a single nucleotide polymorphism in VEGF-A had an independent association with progression-free and overall survival [59].

### 2.3. Pericyte Cell-Related Markers

Pericytes are other tumor-recruited cells in the tumor microenvironment. They attach to the capillary wall and, together with endothelial cells, promote cancer cell intravasation by performing pro-angiogenic effects and contributing to metastases [112].

Various pericyte markers are also associated with mCRC survival. Thus, absence of smooth muscle actin-positive pericytes in primary tumors or different subsets of perivascular cells defined by PDGF receptor-α and -β are associated with liver metastasis and mCRC patients’ survival [60,61].

Pericyte markers are also predictive biomarkers. Thus, low pericyte coverage, measured by alpha-smooth muscle actin expression or pericyte germline polymorphisms, can be used as predictor of bevacizumab-based treatments [62,63].

The use of antiangiogenic agents is widespread in mCRC treatments. Due to the complexity of endothelial cells, pericytes and other stromal cells as well as the overlap between the various angiogenic factors that orchestrate the angiogenesis, the interest towards the validation of the truly prognosis and predictive value of promising biomarkers represents a significant challenge. In this way, large clinical studies are needed to acquire definitive data and to correctly support the clinical decision based on expected benefit effects and just not based on benefit/toxicity effects that are currently largely used [113].

### 2.4. Immune Cell-Related Markers

Recurrence risk in CRC patients needs to be calculated better. The quantification of immune cells based on the consensus Immunoscore criteria reviewed in [114], introduce a new cancer classification with the highest relative contribution among various clinical parameters including the TNM classification system for the evaluation of predicted risk [115]. Similarly, consensus molecular subtypes 1 (CMS1), which correspond to tuors with strong immune cell infiltration and cytotoxic signaling activation, is associated with poor prognosis in metastatic settings [116]. An immune metabolic classification of mCRC into the three different clusters showed its use as a guide to combined immune-metabolic therapies in mCRC [117].

#### 2.4.1. Lymphocytes

The prognostic and predictive value of adaptive immune cells has also been studied in colorectal liver metastases, confirming that T and B cell densities can be considered important clinical tools for mCRC patients’ management. Thus, the quantification of these cells in liver metastasis samples showed the association between increased immune densities and beneficial response as well as a strong predictive value for overall and disease-free survival [64]. The impact of natural killer and T cells infiltrating liver metastases was also seen in patients undergoing hepatectomy after neoadjuvant chemotherapy associated with chemotherapy response and overall survival prognosis in mCRC patients [66]. Similarly, high tumor-infiltrating lymphocytes strongly predict better survival of mCRC patients [65]. Recently, it was observed that primary lesions and synchronous and metachronous metastases have different immune infiltrate and mutational diversity, which belies the assumption that metastases are homogeneous. Thus, the immune phenotype of the T and B cell score at the least-infiltrated metastases had stronger predictive value than at other metastases [67].

Interestingly, a decrease in Treg and an increase in Th17 cells after chemotherapy treatment (5-FU, oxaliplatin), which is associated with poor prognosis, were also observed. However, a decrease in granulocytic myeloid-derived suppressor cells (after 5-FU plus leucovorina and oxaliplatino (FOLFOX)-bevacizumab treatment) is associated with better outcome [68]. Moreover, the identification of the immunoscore could help in the classification of patients and in their eligibility for different immunotherapeutic treatments. For instance, microsatellite instability status and CD8 T-cells are associated with the risk of death. However, the combination of both variables decreased clearly the risk of mortality, which allowed the evaluation of this combined variable, with a view to identifying a subset of patients with a different prognosis and thus adjusting personalized treatment [69]. Although mCRC patients with microsatellite instability-high may show the clinical benefit of immune checkpoint inhibitors, approximately 25% are resistant to these treatments. Therefore, the tumor’s mutational burden and tumor-infiltrating lymphocytes demonstrated a clear association with clinical responses and survival benefit. This showed their potential as predictive biomarkers of immune checkpoint inhibitors in microsatellite instability-high mCRC patients [70]. In contrast, a recent study by Millen et al. did not find any prognostic value for tumor-infiltrating lymphocytes in primary tumors of patients with de novo mCRC. However, the authors identified a subgroup of patients with microsatellite stable tumors and a high increase of tumor-infiltrating lymphocytes/PDL-1, which could be considered for immune checkpoint therapies [118]. 

#### 2.4.2. Macrophages

In recent years, the role and clinical relevance of macrophages in CRC liver metastasis have been subjects of increased attention. In fact, tumor-associated macrophage (TAM) infiltration in resected colorectal liver metastases was independently associated with better outcome [71]. However, contradictory data casting doubt on their involvement in CRC progression and thus on their value as prognostic factors have been reported. Most probably these controversies are due to the high heterogeneity of TAMs. Thus, to decipher this diversity, personalized therapeutic approaches are needed [119]. Variations in genes regulating TAM-related functions were found to be prognostic markers of clinical outcomes in mCRC treated with bevacizumab-containing chemotherapy [72]. Moreover, CCR2^+^ inflammatory monocytes are recruited to the tumor microenvironment by CCL2-expressing neoplastic cells in liver metastases, which confers poor prognosis on mCRC patients [73]. However, a polymorphic region study of the induced nitric oxide synthase (iNOS) gene, a surrogate marker of M1 macrophage activation, failed to assess its prognostic value in mCRC patients [120]. 

#### 2.4.3. Challenges, Limitations and Future Direction of Immune Cell-Related Biomarkers in mCRC

As it will be reviewed below, the list of approved indications and agents to modulate the immune checkpoints is rapidly growing. Although the driving elements that orchestrate the immune response differs among patients and even in different organ site of the same individual, many pivotal characteristics could determine the disease evolution. Thus, it is obvious the need to develop new strategies to identify new prognostic and predictive biomarkers for novel therapeutic strategies and to identify best responding patients.

The high interplay between the tumor and immune cells forces to develop comprehensive assessment of “multi-immune markers” including multiplexed and multimodality biomarkers. Moreover, standardized assays may progress to unify scoring systems by clinicians, for instance regarding PD-L1 expressing cells [121]. The success of immune-based biomarkers will contribute to an optimal sub-classification of the patients for immunotherapies approaches avoiding unnecessary side effect, the high cost of the treatments and searching the particular needs of every patient.

### 2.5. Tumor Microenvironment-derived Markers in Liquid Biopsy

Liquid biopsy as a source of tumor microenvironment components is a minimally invasive method. As such, it is potentially a good, new method for early CRC detection and as a tracking disease biomarker [28]. The level of circulating tumor cells in peripheral blood is an independent predictor of mCRC patients’ outcome [122]. However, recent studies describe new types of circulating non-tumoral cells as well as their derived markers and extracellular matrix components in cancer patients, which have clinical implications for cancer diagnosis, prognosis and treatment response.

#### 2.5.1. Circulating Stromal Cells and Related Markers

##### Circulating Endothelial Progenitors, Circulating Endothelial Cells and Tumor Angiogenic Markers

Several studies indicate the potential role of circulating endothelial cells as surrogates’ prognostic biomarkers in CRC [74]. This prognostic value was recently defined as higher than that related to circulating tumor cells [75].

Importantly, several studies show the predictive value of these circulating cells in bevacizumab-treated mCRC patients. Thus, the absolute number of circulating endothelial cells as well as their progenitors decreased in bevacizumab-treated patients [76,77,78]. Manzoni et al. also observed the prognostic value of circulating endothelial cells, although, in this case, the increase in circulating endothelial cells after the sixth cycle was associated with progression-free survival [79]. Similarly, circulating endothelial cells and those positive for CD276 (tumor-associated endothelial cell marker) significantly increase after combined bevacizumab plus chemotherapy treatment in mCRC and do not have any predictive value of response to first-line treatment [123].

Treatment with bevacizumab monotherapy or with bevacizumab combined with chemotherapy showed angiogenesis growth factors and related cytokine level changes in plasma of mCRC patients. This event was called angiogenic switch [124]. Recently, it was observed that this angiogenic switch is associated with better disease control and longer progression-free survival, suggesting its potential role as a marker of angiogenesis inhibitor effectiveness [80]. Similarly, VEGF-D plasma concentration was described as a potential predictive biomarker for ramucirumab efficacy in second-line mCRC patients in the III RAISE trial [81]. Moreover, genomic DNA isolated from whole blood showed the prognostic value of VEGF-A and ICAM-1 variants in bevacizumab-treated patients [82]. Similarly, VEGF, HGF, EGF and PDGF-AA levels decreased in partial-response mCRC patients under capecitabine and oxaliplatin chemotherapy protocols; and increased HGF levels were found in progressive disease patients, indicating the prognostic information of these angiogenic factors [83]. 

##### Circulating Immune Cells and Inflammation-Related Markers

The systemic immune-inflammation index is calculated by the combined data of platelet, neutrophil and lymphocyte counts [125]. In mCRC patients a high immune-inflammation index is associated with poor clinical outcomes. Use in combination with lymphocyte response may improve prognostic value for these patients [84,85]. This index also behaves as a predictive marker for mCRC patients undergoing first-line chemotherapy with or without bevacizumab. Those patients with low immune-inflammation index in the chemotherapy plus bevacizumab group showed better progression-free survival rates than those treated with chemotherapy alone [86]. In a similar way, a set of inflammatory and angiogenesis-related serum markers, including higher amounts of epidermal growth factor and macrophage-derived chemokine and lower IL-10, IL-6 and IL-8 levels, were observed in responder mCRC patients undergoing irinotecan plus bevacizumab-based treatment [87].

The ratios between different immune cells and between immune cells and platelets are also prognostic and predictive biomarkers in mCRC patients. A decrease in mean platelet volume and in the platelet-to-lymphocyte ratio is associated with worse overall survival in mCRC patients [88,89]. A low platelet-to-lymphocyte ratio in pretreated mCRC patients behaves as a predictor of aflibercept response and is suggested as a possible tool for patient responder selection [90]. Moreover, neutrophil-lymphocyte ratio, platelet-lymphocyte ratio and systemic immune-inflammation index are predictors of good response of cetuximab-combined therapy in mCRC patients [91]. In line with these data, many studies confirm the neutrophil-lymphocyte ratio in mCRC under treatment options such as TAS-102 [92], first-line bevacizumab plus chemotherapy [93,94] and chemotherapy plus cetuximab [95]. Moreover, a high neutrophil-lymphocyte ratio also correlates with a cytokine profile relating to key biological processes involved in carcinogenesis that provides enhanced prognostic information [126]. In contrast, a study from Colloca et al. describes the prognostic value of the neutrophil-lymphocyte ratio for only those patients with left-sided mCRC [127]. However, high levels of the monocyte-lymphocyte ratio are associated with worse outcome in older mCRC patients treated with first-line chemotherapy [96]. 

The circulating T cell lymphocyte subsets are also confirmed as mCRC biomarkers. An analysis of T cell subsets in peripheral blood of mCRC patients under folinic acid, 5-FU and irinotecan (FOLFIRI) plus bevacizumab treatment showed changes in CD4+ and Treg ratios during the treatment; their decrease was associated with better responses and better patient outcome [97]. In a similar way, the levels of Treg and T helper rate are associated with partial response, stable disease or progressive disease, suggesting that this rate has a significant, strong association with therapeutic response [98]. Additionally, measurement of Tregs and CD8+ T cells has prognostic value; patients with right-side mCRC have a favorable outcome in first-line bevacizumab-treated patients [99].

#### 2.5.2. Exosomes and Noncoding-RNAs

Although it is sometimes difficult to assess their specific origin, since they can be released by different cells, including tumor cells, exosomes released by tumor microenvironment cells and their cargo could be used as biomarker tools for oncology clinical practice in mCRC patients [128].

Various efforts have attempted to provide a detailed description of microenvironment-derived exosome composition to identify potential biomarkers in CRC [28]. Thus, different amounts of noncoding RNA regulatory elements were specifically observed by NGS in CAF-derived exosomes that might play a role between CAFs and cancer cells and/or other stromal cells [129]. In the case of miRNAs, transfers of miR-92a-3p by CAF-derived exosomes to tumor cells contribute to cell stemness, epithelial-mesenchymal transition and metastasis, and 5-FU/Oxaliplatin resistance. Moreover, high levels of this miRNA in plasma exosomes are associated with metastatic disease and chemotherapy resistance [100].

#### 2.5.3. Extracellular Matrix-derived Components

Different extracellular matrix components are released into the peripheral blood during tumor development. These components represent potential biomarkers for the diagnosis, prognosis and treatment response of different types of cancers including CRC tumors [130]. Although several studies have analyzed these biomarkers in CRC patients with liver metastases, their diagnostic and prognostic value is not clear. Thus, plasma proMMP-2, -9 and tissular inhibitor of metalloproteinases 1 (TIMP-1) levels showed no diagnostic or prognostic value in mCRC patients [131]. Another biomarker, collagen fragments from extracellular matrix remodeling that are released to circulation, have been studied as diagnostic and prognostic biomarkers in many tumors [132]. However, again, no studies demonstrate clearly their biomarker value in mCRC. For instance, endostatin levels are associated with age, tumor invasion and poor differentiation, but not with metastases in CRC patients [133].

#### 2.5.4. Challenges, Limitations and Future Direction of Biopsy Liquid Biomarkers in mCRC

The implementation of liquid biopsy response-biomarkers into the clinical management of the patients is a powerful technology for diagnosis and mostly for disease evolution monitoring. However, some limitations have been considered. The main issue is the heterogeneity among different patients, even sometimes in the same individual, which complicate the determination of cut-off for biomarkers measurements; and the lack of specific international guidelines and standardized techniques to analyze the big amounts of described biomarkers in liquid biopsy. In this way, it is marked the need to develop larger, prospective and multicenter clinical trials to properly set up the best approaches for mCRC patients’ management.

## 3. Targeting Tumor Microenvironment in mCRC

Metastatic colorectal patients are conventionally treated with chemotherapy based on fluoropyrimidine, oxaliplatin and irinotecan, combined or in sequence with monoclonal antibodies targeting epidermal growth factor receptor or vascular endothelial growth factor [134].

Due to the supportive role of tumor microenvironment in tumor growth, targeting its cells and compounds looks like a good strategy to prevent and treat metastatic disease, including liver metastasis. Thus, targeting angiogenic pathways and regulating the immune response are two widely developed and oft-used research pipelines. 

### 3.1. Antiangiogenic Therapy

As stated above, angiogenesis is essential to liver metastasis growth in colorectal cancer patients. Thus, different angiogenesis-related components, such as VEGF, PDGF, placental growth factor (PlGF) and FGF, as well as their receptors, have been investigated under different treatment settings to assess their safety and efficacy in mCRC treatment [135].

Bevacizumab was the first approved anti-angiogenic used in mCRC patients. It is a humanized monoclonal antibody that binds to VEGF-A and inhibits its binding to the receptor [136]. There are many studies confirming bevacizumab efficacy in mCRC patients, although no benefits were observed in patients with early stages of the disease [135].

The humanized recombinant fusion protein aflibercept also prevents the binding of VEGF-A, VEGF-B, PlGF-1 and PlGF-2 to their respective receptors. A significant survival benefit was observed in mCRC patients previously treated with oxaliplatin [137].

Ramucirumab is another humanized monoclonal antibody that targets the VEFGR2 extracellular domain and prevents binding with its ligand. It has shown benefit against different tumor types including colorectal cancer. It was recently approved by the US Food and Drug Administration (FDA) for use in treating mCRC patients as a second-line therapy in combination with FOLFIRI treatment. Several clinical trials showed a favorable toxicity profile and promising antitumor efficacy in colorectal cancer patients [138]. 

There are also other compounds with multikinase inhibitors function such as regorafenib or nintedanib. Regorafenib targets VEGFR1-3, TIE2, KIT, RET, RAF, PDGFR and FGFR, among other kinases, regulating tumor angiogenesis and tumor microenvironment and showing therapeutic benefit in various malignancies [139]. After phase III trials showed the survival benefit of regorafenib in the refractory mCRC setting [140,141], it was approved by the FDA for the treatment of mCRC after progression on chemotherapy, anti–VEGF therapy or anti–EGFR therapy [142]. However, regorafenib shows some adverse effects such as hand-foot skin reaction, desquamation/rash, hypertension and diarrhea, which usually occur during the first cycle of treatment and decrease over time. Thus, their early recognition and correct management could increase adherence and treatment duration [143]. Finally, nintedanib is also a multikinase inhibitor which targets VEGFR1-3, PDGFR-α/β, and FGFR1-3, as well as RET-proto-oncogene (RET), fms like tyrosine kinase 3 (FLT3), Lck and Lyn. It arrested tumor growth in xenograft solid tumors [144]. Although it showed antitumor activity and maintained an acceptable safety profile in a phase I trial [145], it did not improve overall survival and just a modest increase in progression-free survival occurred in a phase III study with refractory mCRC patients [146]. 

Another compound is VCAM-1 or CD106, which is predominantly expressed in endothelial cells, although under inflammation and chronic conditions it is also expressed on the surface of macrophages, dendritic cells, bone marrow fibroblasts, myoblasts, oocytes, Kupffer cells, Sertoli cells and cancer cells. Considerable evidence associates VCAM-1 and tumor angiogenesis and metastasis. Due to adverse effects or drug resistance in long-term bevacizumab-treated patients, new targets may be identified, with VCAM-1 targeting a possible strategy to inhibit tumor metastasis [147]. 

### 3.2. Immunotherapy

Immunotherapy is widely used in many types of solid cancer, including colorectal cancer patients. However, in CRC patients it only showed benefit in those patients with MSI-H:dMMR tumors [148,149]. There are three monoclonal antibodies approved by the FDA for mCRC patients harboring MSI-H:dMMR molecular profile tumors: pembrolizumab, nivolumab and ipilimumab [150,151,152]. 

#### 3.2.1. T Cells

As stated above, measurement of infiltrated T cells is associated with patient prognosis and even treatment response. The immunity mediated by T cells includes different steps regulated by stimulatory and inhibitory signals that counterbalance the immune response. Usually, inhibitory ligands and receptors involved in cytotoxic T cell regulation, which are commonly known as immune checkpoints, are overexpressed in tumor cells and other non-tumor microenvironmental cells. Blocking these immune checkpoints, and thus targeting lymphocyte receptors or their ligands, enhances endogenous antitumor activity [153]. Most of the immunotherapeutic agents are guided to the MHC-TCR, in which the upregulation of different immune checkpoints, such as PD-1, PD-L1, CTLA-4, indoleamine 2, 3-dioxygenase and lymphocyte-activation gene 3, suppress the cytotoxic activity of T-cells and enhance their regulatory effects [149]. 

PD-1 is expressed in various hematopoietic cell linages and is overexpressed in tumor cells [153]. The interaction between the ligand PD-1 and its receptor PD-L1 promotes polarization of effector T cells to Tregs, decreasing cytokine production and inhibiting proliferation of T cells [154]. Pembrolizumab and nivolumab are two monoclonal antibodies that block PD1 and show benefit in mCRC patients with mismatch-repair-deficient and microsatellite instability-high. Importantly, in a subset of patients, these treatments achieve long-term remission, indicating the huge potential of these therapies in mCRC patients [148]. 

Similarly, CTLA-4 also behaves as an immune checkpoint downregulating tumor-reactive T-cell activation, clonal expansion and subsequent tumor rejection [155]. Ipilimumab, an anti-CTLA-4, showed a lasting clinical benefit in combination with nivolumab in mCRC patients with mismatch-repair-deficient and microsatellite instability-high [156].

Despite the benefit of immune checkpoint inhibitors in patients with dMMR-MSI-H phenotype, a subset of patients does not respond to these treatments. Moreover, 95% of mCRC patients have mismatch repair-proficient-microsatellite instability-low (pMMR-MSI-L) phenotype, for which the approved therapies showed unsuccessful results [157]. Thus, in order to improve mCRC patients’ outcome, ongoing studies focus on biomarker identification to predict good response to different treatments, as well as to introduce combined therapies with high standards of biological care, anti-angiogenic agents and chemotherapy regimens [158]. Moreover, different studies in pMMR-MSI-L patients have investigated the manipulation of the immune response, studying the efficacy of immunotherapy in identifying optimal agent combinations in this mCRC phenotype, which is the most common and thus important. In this context, combining immune checkpoint blockade with other immune-modulating agents could enhance T-cell activation and infiltration into the tumors. These combinations include immunotherapy with radiotherapy, chemotherapy or antiangiogenic agents, as well as targeted therapy, tumor vaccines, adoptive cell transfer, bispecific T-Cell engaging antibody therapy, indoleamine 2,3-Dioxygenase 1 Inhibitors, epigenetic modulators and depletion of T-Regs and Myeloid-derived suppressor cells [158].

#### 3.2.2. Macrophages

Macrophages in the tumor microenvironment are polarized in M1- or M2-like phenotypes. In the M1 phenotype, a classic activated phenotype, they exert antitumor activities including direct killing of tumor cells, production of pro-inflammatory cytokines, and greater antigen presentation abilities. In the M2 phenotype, the macrophages orchestrate an inflammatory microenvironment inducing angiogenesis, matrix deposition and tissue remodeling, which lead to tumor cell growth, immune suppression and metastatic process enhancement [119]. 

In addition, TAMs in the tumor microenvironment modify the efficacy of anticancer strategies, including treatments for colorectal liver metastasis in which targeted therapies are commonly used [159]. Conventional chemotherapy and radiotherapy treatments cause tissue damage, inducing a tissue repair response and an inflammatory environment in which the general context, including tumor immunogenicity, tissue of origin, and microbial conditions, defines the contribution or limitation of macrophages to the successful chemotherapy agent. Similarly, the interplay of immune response and TAM-proangiogenic activity influences the results of targeted and anti-angiogenic therapies [160]. In immunomodulatory therapies, macrophages express the ligands for PD-1 and for CTLA-4 as B7H4, PD-L1, PD-L2 or B7-1 and B7-2, respectively. Therefore, macrophages are strongly involved in checkpoint blockage-based therapies.

On the basis of these data, different therapeutic strategies are under development to deplete TAMs or use them as weapons to eliminate cancer. With TAM reprogramming immunosuppressive and pro-tumoral macrophages can be converted into immunostimulatory and anti-tumor cytotoxic effector cells. Thus, different approaches have been developed to polarize M2-macrophages into M1-macrophages and thus promising anti-tumor therapies. These strategies include: editing of the TAM genome by the inhibition of immunosuppressive genes with clustered regularly interspaced short palindromic repeats/Cas9 (CRISPR/Cas9) or lentiviral vector; nanoparticles packing Small interfering RNA (siRNAs), miRNAs or mRNAs to regulate gene transcription; Toll-like receptors and Stimulator of interferon genes agonists; monoclonal antibodies targeting CD47/SIRPα axis, activating CD40 or blocking the macrophage receptor with collagenous structure (MARCO) receptor; stimulation of pattern recognition receptors by classical chemotherapy agents or low doses of radiation; and manipulation of TAM metabolism [161]. 

It was observed in a murine model, in relation to metastatic CRC, that targeting CCRr reduces TAM accumulations in liver metastases, restoring anti-tumor immunity and improving chemotherapy responses [73]. Moreover, macrophage repolarization occurs in patients under CCR5 blockage therapies, showing anti-tumoral effects in a phase I trial with refractory mCRC patients [162]. Furthermore, a multi-institutional retrospective study including mCRC patients treated with GOLFIG regimen (gemcitabine + FOLFOX with low-dose of recombinant interleukin-2 and granulocyte-macrophage colony stimulating factor) confirmed the superiority of this treatment over standard FOLFOX regimens [163].

### 3.3. Targeting Cancer-Associated Fibroblasts and Extracellular Matrix

#### 3.3.1. Cancer-Associated Fibroblasts and TGF-β

As stated above, CAFs are emerging as the main enhancers of tumor progression. Therefore, targeting either CAFs or TGF-β signaling in CAF-mediated cancer progression, as well as the ECM, which is mainly produced by fibroblasts, is considered a promising anti-cancer strategy. However, limitations in our understanding of CAF origin and CAF functional heterogeneity might represent difficulties in CAF targeting for a therapeutic benefit [102]. Several studies of various cancers, including mCRC, showed antitumoral effects when the crosstalk between CAFs and cancer stem cells is targeted by depletion of CAF subpopulations or interference with the activation of signaling pathways of cancer stem cells [164].

FAP is a cell surface serine protease which has been proposed as a CAF marker although it may be expressed by some epithelial tumor cells and other TME cells [165]. Recent studies have associated FAP expression in CAFs with specific immune features in colon cancer, suggesting a mechanism of fibroblast-immune crosstalk and opening up a new field for cancer treatment strategies [166]. Along these lines, depletion of FAP+ fibroblasts by FAP-targeted chimeric antigen receptor (CAR) T cells disrupts tumor-promoting desmoplasia by reducing extracellular matrix proteins and glycosaminoglycans [167]. Moreover, and probably because the current biomarkers used to determine drug efficacy are not CAF-specific, targeting FAP with talabostat and sibrotuzumab drugs showed minimal clinical activity in previously treated mCRC patients [168,169,170]. 

Interestingly, several nanoparticle formulations for delivering drugs to hepatic stellate cells/CAFs and reprograming them as CAFs were tested successfully. These have become attractive targets for cancer therapies, especially in the liver [171]. 

TGF-β is a cytokine that plays a major role in tumorigenesis. TGF-β regulates cell growth and differentiation, apoptosis, cell motility, extracellular matrix production, angiogenesis and cell immune responses. Interestingly, it is involved in tumor suppression during early stages of tumor development but acts as a potent enhancer of tumorogenesis during later stages, when it promotes tumor growth and regulates stromal cells, which generates a permissive microenvironment for tumor invasion and metastases. Thus, TGF-β activates tumor angiogenesis and CAFs as well as inducing immunosuppression [168]. Interestingly, gene expression profiles showed that TGF-β signaling is the most significant gene pathway in liver metastases of colorectal cancer [169].

As one of the main players in cancer development, several TGF-β signaling inhibitors have been developed to target its oncogenic properties. Therefore, antisense oligonucleotides, receptor kinase inhibitors and neutralizing antibodies have been studied in different preclinical models [168]. However, due to the complexity and pleiotropic nature of TGF-β, its targeting continues to be a challenge and requires careful administration/dosing of its therapies as well as patient selection to overcome on-target and off-target toxic side-effects [170].

Antisense oligonucleotides are short molecules of 13–25 nucleotides that downregulate TGF-β synthesis by interfering with mRNA function. There are several molecules such as AP11014 and AP15012 in preclinical development to treat tumors including CRC [171]. 

Of kinase inhibitors, LY2109761 inhibits the activation of Smad and non-Smad pathways mediated by TGF in colon adenocarcinoma cells by attenuating cell migration, invasion and tumorigenicity of cancer cells. Moreover, in a preclinical experimental metastasis model, this agent also decreased liver metastases and prolonged survival, indicating its therapeutic potential for metastatic colorectal cancer patients [172]. 

Several monoclonal antibodies such as 2G7, 1D11, lerdelimumab (CAT152), metelimumab (CAT152) and fresolimumab (GC1008) also neutralize the different isoforms of TGF-β [171,173,174]. Fresolimumab demonstrates acceptable safety and antitumor activity in melanoma and renal cell carcinoma, but no data exist for CRC patients [175].

Finally, as mentioned above, TGF-β is involved in immunosuppression. Therefore, targeting PD-1 with TGF-β inhibitors is a biological rationale for cancer treatment. Various trials exploring combinations of PD-1/PD-L1 inhibitors are under development [171].

#### 3.3.2. Extracellular Matrix

ECM is a dynamic structure that modulates and regulates cell functions relating to cancer promotion, such as adhesion, migration, proliferation and differentiation. This regulation is mediated by the remodeling, production and degradation of its components [172]. MMPs, a family of proteolytic enzymes, are directly involved in ECM degradation and thus in tumor invasion, neoangiogenesis and metastasis. Accordingly, different agents have been developed with MMPs as therapy targets [173].

Small molecules that target the zinc ion at the active site of MMPs were the first generation of MMP inhibitors. Similarly, small molecules that target enzyme exosites were also designed [174]. However, these molecules, such as hydroxamate, marimastat or rebimastat, are unstable in part, lack selectivity in targeting the catalytic domain of proteases and failed in late stages of the clinical trials [175]. 

Another type of ECM inhibitor focuses on collagens. Thus, triple helical peptides that mimic the triple helical collagen substrate of MMPs were developed to target different domains of MMPs [174]. 

Various anti-MMP-9 strategies were also developed by using several monoclonal antibodies. The humanized anti-MMP-9, andecaliximab, showed safety and moderate effectiveness in gastric cancer patients when combined with classic cytotoxic drugs [176]. The monoclonal antibodies anti-MMP-9, AB0041 and AB0046, inhibit tumor growth and metastasis in a preclinical colorectal cancer model [177]. 

### 3.4. Other Targets

#### 3.4.1. Lipopolysaccharide

The anatomical location of the colon means it has the largest number of microbes. Thus, CRC develops in close association with fecal bacteria and the gut microbiome, which are directly related with cancer progression [178]. An in-depth characterization of the interaction between the intestinal microbiota and mucus in healthy and CRC subjects is reviewed in [179]. High lipopolysaccharide levels, produced by Gram-negative microbiota, are found in CRC patients [180]. Lipopolysaccharide, as an immune-stimulatory ligand, is involved in intestinal inflammation and CRC progression, including liver metastasis promotion [181,182]. Recent studies have shown gut microbiome influence on the efficacy of PD-1-based immunotherapy in melanoma patients [183]. 

Thus, a recent study demonstrated the prevalent role of lipopolysaccharide in CRC immunotherapy. The authors developed a nanoparticle-based lipopolysaccharide trap system to promote PD-1-based therapies for CRC and inhibit liver metastases [184]. 

#### 3.4.2. Nitric Oxide (NO)-NO Synthases (NOS)

NOS produce the free radical gas NO, which exerts dual effects on cancer as a pro- and anti-tumor agent. In CRC, NO is involved in many signaling pathways; and various agents have been developed for CRC therapy with clinical benefits. However, well-designed clinical studies to adjust therapeutical strategies and develop more potent and less toxic therapies are still needed for CRC patients’ treatment [185]. 

## 4. Conclusions

Colorectal cancer accounts for approximately 10% of all annually diagnosed cancers and cancer-related deaths worldwide [186]. Metastasis is the most common cause of death in patients with CRC and the mechanisms underlying this process remain poorly understood. Early detection of the disease and prognostic and treatment response biomarkers have been one of the main focuses of studies in recent decades. 

The interplay between tumor and stromal cells in the tumor microenvironment and at distant locations are rising in importance as potential mechanisms of the tumor progression. Therefore, it is crucial to identify the events that contribute to the preparation of a favorable pre-metastatic niche, involving local stromal cells, bone marrow-derived cells, immune cells, tumor-derived secreted factors and extracellular vesicles. In this line, recent insights relating to the capacity of tumor microenviroment cells and liquid biopsy biomarkers for both patient prognosis and treatment response prediction are emerging as the new biomarkers for metastatic colorectal cancer. Moreover, given the supportive role of tumor microenvironment in the tumor growth, targeting its cells and compounds seems a good strategy to prevent and treat metastatic disease. Thus, clinical strategies to tackle metastatic disease based on the targeting of angiogenic pathways and regulating the immune response are two research approaches emerging as novel and relevant therapies for CRC patients’ treatment.

## Figures and Tables

**Table 1:** Mediators of microenvironment as enhancers of mCRC progression.

Molecule	Role	References
VEGF	Stimulate migration of myeloid cells to metastatic niche	[9]
	Angiogenesis	[3] [10] [8]
	Chemoattractant for macrophages (VEGFA)	[3]
	Stimulate lymphangiogenesis (VEGFA, C, D)	[3] [8] [18]
TNF-α	Stimulate migration of myeloid cells to metastatic nice	[9] [10]
	Pro-inflammatory	[9] [10]
	Extravasation	[6]
	Immunosuppression	[5]
TGF-β	Stimulate migration of myeloid cells to metastatic nice	[9]
	TME remodeling	[11]
	Pro-inflammatory	[9] [10]
	Macrophage polarization to M2 phenotype	[5]
	Fibroblast activation	[10,19]
CXCL5	Liver metastasis	[11]
CCR6	Liver metastasis	[11]
	Tumor cell recruitment	[16]
EGF	TME remodeling	[11]
IGF	TME remodeling	[11]
Glutamate	Liver metastasis	[12]
ICAM and VCAM	Cell adhesion	[6]
	Extravasation	[6]
MMP	TME remodeling (MMP-2, -9, -13)	[11]
HIF-1	Formation of pre-metastatic nice	[9]
	Extravasation	[8]
CXCR4	Local tumor expansion	[3]
	Extravasation	[3]
CXCL2 CXCL3 CXCL8	Hepatic metastasis (CXCL2)	[3]
	Promote tumor vascularization	[3] [11]
CTLA-4	Immunosuppression	[5]
	Macrophage polarization to M2 phenotype	[5]
PD-1	Immunosuppression	[5]
PDL-1	CD8+ T cell suppression	[9]
CXCL1 CXCL2	MDSC recruitment	[5]
	T-cell inhibition	[5]
CCL5	Liver metastasis	[3]
	T-cell inhibition	[5]
ROS	T-cell inhibition	[5]
ARGINASE	T-cell inhibition	[5]
IL2	Immunosuppression	[5]
	Macrophage polarization to M2 phenotype	[5]
IL10	Immunosuppression and macrophage polarization to M2 phenotype	[5]
IL6	Promote extravasation from lymphatic vessels	[8,20]
	Inflammation	[8]
CCL20	Liver metastasis (attraction of CCR6+ tumor cells)	[16]
CEA	Liver metastasis	[17]
INTEGRINS	Cell adhesion	[6]
	Liver metastasis (α6β1, α6β4, α2β1, αvβ6, αvβ5)	[15] [10] [21]
OSTEOPONTIN	Liver metastasis	[15]

**Table 2:** Prognostic and predictive biomarkers in the tumor microenvironment of mCRC.

	Clinical Association	Reference
**Cancer Associated Fibroblasts**		
PDGFR family, podoplanin, FAP	Association with patients’ survival	[37,38,39]
Endoglin	Poor metastases-free survival	[40]
CXCL1	Decreased overall survival in stage IV CRC patients	[41]
↓ PTEN expression	Worse prognosis in mCRC patients	[42]
↓ miR-198	Associated with worse prognosis	[43,44]
Specific proteomic signature of extracellular matrix proteins	Diagnostic and prognostic value	[45]
Claudin-2	Predictive value in chemotherapy-based treated patients	[46]
Mutation in FGFR1/PDGFRα	Primary resistance mechanism to EGFR blocking antibodies	[47]
CMS4 mesenchymal tumors	Resistance to anti-EGFR agents	[48,49]
FGFR2 amplification	Predictive marker of regorafenib sensitivity	[50]
Interactome signature	Associated with relapsed and refractory patients	[51]
**Endothelial cells**		
Microvessel density	Associated with poor prognosis. Bevacizumab response biomarker	[52,53]
↑ VEGF	Better overall and progression free survival in bevacizumab treated patients	[54]
VEGF-A	Associated with the survival of mCRC patients	[55,56]
VEGFA145b isoform	Resistance to bevacizumab	[57]
↓ NOTCH1 receptor	Predictive value in bevacizumab-treated mCRC patients	[58]
Single nucleotide polymorphism in VEGF-A	Association with survival	[59]
**Pericytes cells**		
↓ smooth muscle actin-positive pericytes	Association with liver metastasis and with the number of metastases	[60]
PDGFR-α/-β	Independent factor for survival of mCRC patients	[61]
↓ pericyte coverage	Predictor of bevacizumab benefit	[62]
Pericyte germline	Clinical outcome of mCRC patients treated with FOLFIRI+bevacizumab	[63]
**Lymphocytes**		
Quantification of T and B cells	Predictive value	[64,65]
Natural killer and T cells infiltrating	Chemotherapy predictive value	[66]
T and B cell score at the least-infiltrated metastases	Strong predictive value than other metastases	[67]
↓ Treg and ↑ Th17 cells	Association with poor prognosis	[68]
↓ granulocytic myeloid-derived suppressor cells	Association with better outcome	[68]
Microsatellite instability status and CD8 T-cell	Association with the risk of death	[69]
Tumor mutational burden and tumor infiltrating lymphocyte	Predictive value in microsatellite instability-high mCRC patients	[70]
**Macrophages**		
↑ TAM infiltration	Association with better outcome	[71]
Gene expression signatures in TAM	Prognostic markers	[72]
CCR2^+^	Association with poor prognosis	[73]

Footnotes: ↓ Downregulation/low levels; ↑ Upregulation/high levels.

**Table 3:** Tumor microenvironment-derived markers in liquid biopsy of mCRC.

	Clinical Association	Reference
**Circulating endothelial progenitors, circulating endothelial cells and tumor angiogenic markers**		
Circulating endothelial cells	Prognostic biomarkers in CRC	[74,75]
Number of circulating endothelial cells and progenitors	Decreased in bevacizumab-treated patients with radiological response	[76]
↓ circulating progenitor and endothelial cells	Association with longer progression-free survival and overall survival of bevacizumab combination chemotherapy treated mCRC patients	[77,78]
Circulating endothelial cells	Association with survival in bevacizumab-based first-line treated patients	[79]
Angiogenic switch	Association with progression free survival	[80]
VEGF-D plasma concentration	Predictive value for ramucirumab efficacy	[81]
VEGF-A and ICAM-1 variants	Prognosis value in bevacizumab treated patients	[82]
VEGF, HGF, EGF, and PDGF-AA levels	Predictive value in chemotherapy-based treated patients	[83]
**Circulating immune cells and inflammatory related markers**		
↑ immune-inflammation index	Association with poor clinical outcomes	[84,85]
Immune-inflammation index	Predictive marker for mCRC patients	[86]
↑ EGF, macrophage-derived chemokine and ↓ IL-10, IL-6 and IL-8 levels	Predictive value in irinotecan/bevacizumab-based treatments	[87]
↓ mean platelet volume and in the platelet-to-lymphocyte ratio	Association with worse overall survival	[88,89]
↓ platelet-to-lymphocyte ratio	Predictor of aflibercept response	[90]
Neutrophil-lymphocyte ratio, platelet-lymphocyte ratio and systemic immune inflammation index are	Predictors oof cetuximab-combined therapy	[91]
Neutrophil-lymphocyte ratio	Predictor of good response	[92,93,94,95]
↑ monocyte-lymphocyte ratio	Worse outcome	[96]
↓ T cells subsets	Association with better outcome	[97]
Levels of Treg and T helper rate	Association with therapeutic response	[98]
Quantification of Tregs and CD8+ T cells	Prognostic value	[99]
**Exosomes and noncoding-RNAs**		
miR-92a-3p	Association with metastatic status and chemotherapy predictive value	[100]

Footnotes: ↓ Downregulation/low levels; ↑ Upregulation/high levels.

## Abbreviations

5-FU	5-fluorouracile
aSMA	Alpha-smooth-muscle actin
BMDCs	Bone marrow-derived dendritic cells
BRAF	Type B rapidly accelerated fibrosarcoma gene
CAFs	Cancer-associated fibroblasts
CAM	Cell-adhesion molecules
CAR	Chimeric antigen receptor
CCL20	C-C Motif Chemokine Ligand 20
CCR6	C-C Motif Chemokine Receptor 6 ()
CD	Cluster of Differentation
CEA	Carcinoembryonic antigen
CMS	Consensus molecular subtypes
CRC	Colorectal cancer
CRISPR/Cas9	Clustered regularly interspaced short palindromic repeats/Cas9
CTLA-4	Cytotoxic T-Lymphocyte Antigen 4
CXCL5	C-X-C motif chemokine 5
CXCR4	C-X-C chemokine Receptor type 4
DCs	Dendritic cells
dMMR	Deficient mismatch repair
ECM	Extracellular matrix
EGF	Epidermal Growth Factor
FACS	Fluorescence-activated cell sorting
FAP	Fibroblast-activated protein
FDA	Food and Drug Administration
FGFR1	Fibroblast growth factor receptor 1
FOLFIRI	Folinic acid, 5-FU and irinotecan treatment
FSP1	Fibroblast-specific protein-1
HER2	Human EGF receptor 2 gene
HGF	Hepatocyte Growth Factor
HIF-1	Hypoxia-inducible Factor 1 ()
ICAM	Intercellular cell-adhesion molecules
IGF	Insulin-like growth factor ())
ILs	Interleukins
iNOS	Induced nitric oxide synthase
KRAS	Kirsten rat sarcoma viral gene
MARCO	Macrophage receptor with collagenous structure
mCRC	Metastasic colorectal cancer
MDSCs	Myeloid-derived supressor cells
MHC	Major histocompatibility complex
miRNAs	MicroRNAs
MMP	Matrix metalloproteinases
MMR	Mismatch repair
mRNA	Messenger RNA
MSI-H	Microsatellite instability-high
NGS	Next generation sequencing
NKs	Natural killer cells
NOTCH1	Neurogenic locus Notch Homolog protein 1
NRAS	Neuroblastoma rat sarcoma viral gene
NTRK	Neurotrophic receptor tyrosine kinase gene
PD-1	Programmed Death-ligand 1
PDGF	Platelet-derived growth factor
PDGFR	Platelet-derived growth factor receptor
PIK3CA	Phosphatidylinositol 3-kinase catalytic subunit alpha gene
PlGF	Placental growth factor
pMMR-MSI-L	Mismatch repair-proficient-microsatellite instability-low
PTEN	Phosphatase and tensin homologue
RAS	Rat sarcoma oncogene
ROS	Reactive Oxygen Species
siRNAs	Small interfering RNA
TAM	Tumor-associated macrophage
TGF-β	Transforming Growth Factor β
TIMP-1	Tissular inhibitor of metalloproteinases 1
TME	Tumor microenvironment
TNFα	Tumor Necrosis Factor α
TNM	Tumor, Node, Metastases
TRAIL	TNF-Related Apoptosis Inducing Ligand
Tsg101	Tumor susceptibility gene 101
VCAM	Vascular cell-adhesion molecules
VEGF	Vascular Endothelial Growth Factor
